# Response to ‘Remarks on the article of Hadas et al.: Transmission of chimeric HIV by mating in conventional mice: prevention by pre-exposure antiretroviral therapy and reduced susceptibility during estrus’

**DOI:** 10.1242/dmm.014167

**Published:** 2014-02

**Authors:** Mary Jane Potash, Eran Hadas, David J. Volsky

**Affiliations:** Molecular Virology Division, St Luke’s-Roosevelt Hospital Center, Columbia University Medical Center, New York, NY 10019, USA

We recognize the concerns raised in this Correspondence (Andersen and Politch, 2014) regarding the article by Hadas et al. (Hadas et al., 2013) and are responding to alleviate them.

(1) The first issue raised is that EcoHIV-infected male mice might transmit virus to females by biting or other casual contact rather than by mating. This is refuted in principal in a definitive study on the routes of horizontal ecotropic retrovirus transmission in mice (Portis et al., 1987). Portis et al. reported that male-to-female transmission in mice occurred essentially exclusively by mating. They obtained corroborative evidence in finding virus in uterine horns within hours of mating. A virological experiment provided conclusive evidence that vaginal transmission is the only retrovirus transmission route from infected males to females. AKR/J females, although susceptible to WM-E retrovirus infection by injection, were completely resistant to transmission from infected males. This resistance was attributed to viral interference, because females express large amounts of Akv envelope in the reproductive tract and WM-E and Akv belong to the same interference group (McAtee and Portis, 1985). Thus, Portis et al. demonstrated that casual contact between infected males and females during caging together does not transmit retrovirus infection. Studies showing sexual transmission of viruses have followed this definitive paper, echoing the common view that caging infected and uninfected mice results in virus transmission by mating by the male-to-female (Jones et al., 2012; Okada et al., 1998) and female-to-male (François et al., 2013) routes.

As a minor point, in Hadas et al., EcoHIV sexual transmission was not confined to mating infected “aggressive” C57BL/6 males to females as suggested in the Correspondence, but also observed in mating outbred *Foxn1^nu^* males to females (figure 4 in Hadas et al., 2013).

(2) The Correspondence cites biological differences in coitus between humans and rodents. One concern raised is that semen is deposited in the uterus in rodents. Although semen rapidly enters the uterus after insemination in rodents, it is deposited in the vagina, as observed for humans (Carballada and Esponda, 1997; Suarez and Pacey, 2006). The Correspondence notes that the mouse penis is barbed and might cause abrasions to the female reproductive tract during coitus. A detailed histological study of the mouse penis describes “spines whose appearance resembled the filiform papillae on the tongue”, a feature we believe is unlikely to cause abrasions (Murakami, 1987).

**Fig. 1.**
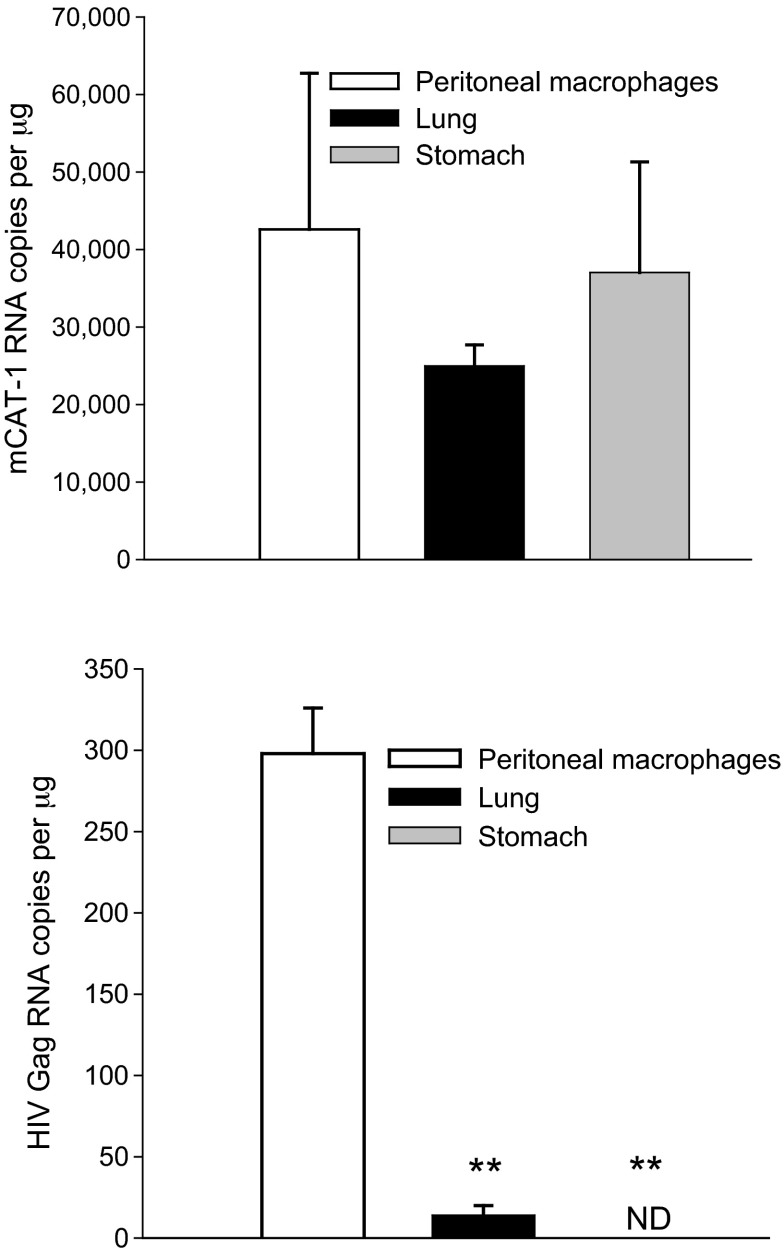
**Mice were infected with EcoHIV by intraperitoneal injection of 10^6^ pg HIV p24**. Tissue RNA was isolated and qPCR for HIV *Gag* and glyceraldehyde phosphate dehydrogenase was conducted as described (Hadas et al., 2013). Murine *CAT-1* cDNA was amplified by qPCR according to the manufacturer’s instructions using a kit (cat. # 4331182) from Life Technologies, Grand Island, NY, USA. Upper panel: *CAT-1* expression; lower panel: HIV *Gag* expression. ND, not detected. **P≤0.01 by Student’s *t*-test.

The Correspondence suggested that EcoHIV tropism in mice is different than HIV tropism in humans because the murine cellular receptor for EcoHIV entry, CAT-1, is widely distributed among tissues. EcoHIV tropism to mouse cells is likely to be conferred not only by the cell surface receptor but also by the activity of the viral long terminal repeat (LTR), as is common for retrovirus replication in mice (Celander and Haseltine, 1984); EcoHIV encodes the HIV LTR. To illustrate this EcoHIV tropism here, we show *CAT-1* and HIV *Gag* RNA in tissues from two C57BL/6 mice 10 days after EcoHIV infection (Fig. 1). CAT-1 is highly expressed in peritoneal macrophages, lung and stomach (upper panel). Although necessary, expression of CAT-1 is insufficient to confer susceptibility to EcoHIV replication. Peritoneal macrophages were productively infected and expressed HIV Gag but neither lung nor stomach was susceptible (Fig. 1, lower panel) (*P*≤0.01).

(3) The Correspondence raised a concern that exposure of females in estrus to EcoHIV-infected males for 1 night is not comparable to exposing unsynchronized females to infected males for several nights. For a comparator, see figure 3B in Hadas et al. showing virus burden after 1 night exposure of unsynchronized females, where all placebo-treated females acquired infection (Hadas et al., 2013). We also wish to clarify that mouse mating is not limited to estrus but also occurs in proestrus or metaestrus (Bronson et al., 1968).

All animal models of human processes have shortcomings. Other animal models of HIV sexual transmission in humans employ administration of cell-free virus stock to hormone-treated anesthetized, immobilized females sometimes treated with vaginal irritants and sometimes treated with fire-polished pipettes repeatedly inserted into the vagina. Our demonstration of EcoHIV transmission by coitus in mice can provide a foundation for further model development and prove to be valuable in understanding the primary transmission route of HIV in humans to better control or prevent it.
